# Considering Culinary Use of Lavender: Sensory and Cultural Factors

**DOI:** 10.3390/foods15193572

**Published:** 2026-10-08

**Authors:** Ceyhun Uçuk, Charles Spence

**Affiliations:** 1 Tourism Faculty, Gastronomy and Culinary Arts Department, Gaziantep University, Gaziantep 27100, Türkiye; ceyhunucuk@gantep.edu.tr; 2 Department of Experimental Psychology, University of Oxford, Life and Mind Building, South Parks Road, Oxford OX1 3EL, UK

**Keywords:** lavender, Mediterranean herbs, anxiolytic, linalool, anti-microbial hypothesis

## Abstract

Lavender is a Mediterranean herb that is used relatively infrequently in the kitchen compared to other Mediterranean herbs, despite growing interest in its culinary applications. The question to be addressed in this narrative historical review is why that should be so. Several alternative hypotheses (historical, chemical, perceptual, psychological, physiological, and antimicrobial) are outlined and critically evaluated. Ultimately, it is suggested that lavender’s longstanding use as an anxiolytic (volatile) stimulus and its enduring association with fragrance, relaxation, and sleep may elicit cognitive inconsistency (or dissonance) with the notion of its use in the food and drink we consume (at least when perceptible as lavender, rather than when blending with other ingredients), while differences in antimicrobial activity, availability, and preservation appear insufficient to explain its comparatively limited culinary role. At the same time, lavender’s growing use in contemporary food and drink suggests that this association is neither fixed nor necessarily prohibitive, with culinary acceptance likely depending on context, familiarity, cultivar, concentration, and how its distinctive flavour is incorporated into food and drink.

## 1. Introduction

Amongst the so-called Mediterranean herbs, such as rosemary, thyme, oregano, marjoram, sage, mint, and lavender, the latter is unusual in that it is rarely used in a culinary setting [1] (and for those wondering why basil does not appear in this list, that is because it is a relatively recent addition to Mediterranean cuisine, only really being popularised from the end of the 19th Century in Northern Italian cuisine [2]). In this narrative historical review [3], we ask why that might be so. Is there something in the flavour profile of lavender (perhaps the principal anxiolytic compound, linalool) that simply does not work well with other ingredients/flavours (i.e., ref. [4])? Or, thinking in terms of the anti-microbial hypothesis [5,6,7], might it simply be that lavender fails to provide the kinds of anti-microbial benefits that other Mediterranean herbs and spices deliver? Or could it be that lavender’s historical use to relax people, and aid sleep [8], means that people conceptualise it in a way that is somehow incongruent with its use in the kitchen?

According to Guo and Wang [9] (p. 1), “Lavender and its products have excellent flavor properties.” (Similarly, Elsharif, Banerjee, and Buettner [10] (p. 1) describe linalool, one of the principal volatiles in lavender, as having ‘a pleasant odour’.) However, besides its putatively appealing sensory attributes, lavender has received a lot of attention for its antibacterial, antioxidant, and preservative properties, being a promising natural ingredient for various food applications [11,12]. Due to its pleasant aroma and wide range of biological activities, lavender essential oil has shown potential as a natural flavouring and preservative in a variety of food products [13,14]. Reflecting this growing culinary interest, lavender has been described as a versatile modern food ingredient, one that can be used as a flavouring agent in desserts, baked goods, chocolates, ice creams and cocktails, as a seasoning in savoury Mediterranean dishes, herbal teas, infused honeys, oils and artisanal confections [15]. Nevertheless, it is striking that these apparently attractive sensory qualities have seemingly never resulted in lavender reaching the full culinary popularity of other Mediterranean herbs, such as rosemary, thyme, oregano, or sage. This peripheral culinary status is likewise evident in initial psychological studies: an investigation into similarity assessments and recognition memory for prevalent culinary herbs and spices excluded lavender from the subjects examined [16].

### 1.1. Review Methodology

The present study used an interdisciplinary narrative inquiry since the research problem required conceptual analysis, rather than the accumulation of homogeneous factual data. Narrative inquiries are particularly suited to addressing complex problems that draw on cross-disciplinary knowledge and aim to develop conceptual understanding rather than to predict the effects of interventions [3,17,18,19].

A structured literature search was undertaken using Scopus, Web of Science, PubMed, and Google Scholar, covering publications from database inception to 23 September 2026, the date of the last search (the literature cut-off). No lower date limit was applied to historical, herbal, and culinary texts, which were identified through citation tracking and targeted searches rather than via database queries, as recommended for improving the transparency and reproducibility of narrative reviews [17]. The search terms included combinations of the following: lavender, lavandula, culinary herbs, gastronomy, flavour chemistry, volatile chemicals, aromatherapy, ethnobotany, sensory perception, antimicrobial action and Mediterranean food. Additional relevant papers were located by backward and forward citation tracking [20], a generally recommended approach for ensuring full coverage of significant material. When exploring the culinary uses of lavender, the peer-reviewed English-language scholarly literature, historical culinary texts, and authoritative review articles were included as long as they contributed directly to the explanation. Studies that were not connected with the aims of the review or were not sufficiently conceptually relevant were removed, following accepted advice for conducting narrative and other non-systematic literature reviews [17,18].

**Figure 1:**
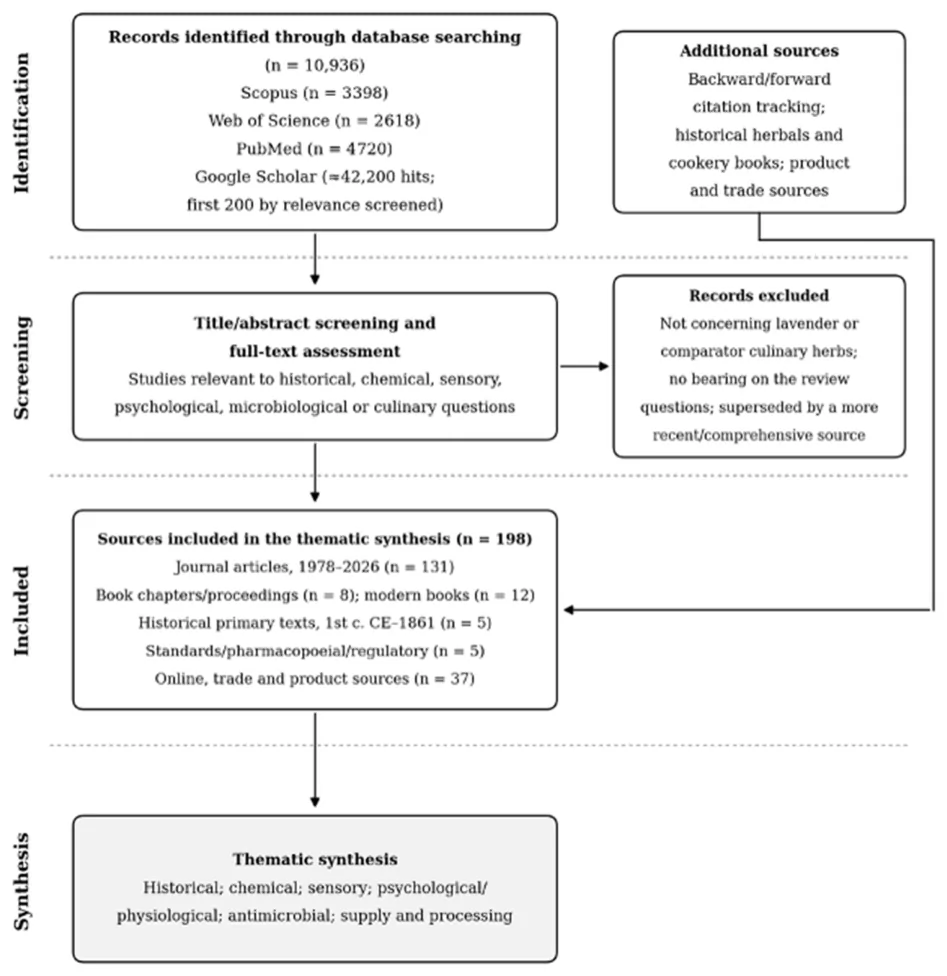
Flow diagram of the literature search and selection process adopted in the present narrative review, showing the number of records identified in each database, the screening and exclusion criteria applied, the additional sources identified through citation tracking and targeted searches, and the number and type of sources included in the thematic synthesis. Search period: database inception to 23 September 2026.

The database searches retrieved 10,936 records (Scopus, n = 3398; Web of Science, n = 2618; PubMed, n = 4720; Google Scholar, approximately 42,200 hits, of which the first 200 results sorted by relevance were screened). Records were screened by title and abstract, and full texts were retrieved for studies relevant to the historical, chemical, sensory, psychological, microbiological, or culinary questions addressed in this review. Records were excluded when they did not concern lavender or the comparator culinary herbs, or when they had no bearing on these questions (for instance, purely agronomic, veterinary, or pharmaceutical-formulation studies); where several studies reported the same finding, the most recent or most comprehensive source was preferred. Additional sources were identified through backward and forward citation tracking and through targeted searches of historical herbals, cookery books, and product and trade sources. In total, 198 sources were included in the thematic synthesis: 131 journal articles (published between 1978 and 2026), 8 book chapters or conference proceedings, 12 modern books, 5 historical primary texts (from Dioscorides’ *De materia medica* to Beeton’s *Household Management* of 1861), 5 international standards, pharmacopeial and regulatory documents, and 37 online, trade, and product sources (Figure 1). Consistent with the narrative design of the review, no formal risk-of-bias assessment or quantitative synthesis was undertaken; these figures are reported to document the search process transparently, rather than to imply the exhaustiveness of a systematic review.

Instead of organising the research in chronological order, or by field, the gathered evidence was thematically synthesised based on the primary explanations arising from the literature regarding lavender’s restricted culinary application. This analytical approach enabled the integration of historical, ethnobotanical, chemical, sensory, psychological, microbiological, and gastronomic data to give a holistic understanding of the factors that may have contributed to the relative absence of lavender from culinary applications, and to identify fruitful areas for future research [3,19]. Figure 1 provides an overview of the review methodology and literature selection process adopted in the present study. The illustrative dish images in Figure 1 were generated with Gemini 1.5 Flash; all chemical structures, compositional data, and textual content were prepared and verified by the authors.

### 1.2. Historical Uses of Lavender

Wild and cultivated vegetables, herbs, and spices were widely used in Greek Antiquity [21]. Researchers have often drawn attention to the fact that in the ancient Mediterranean world, the line separating food from medicine was frequently blurred, since aromatic herbs fulfilled gastronomic, therapeutic, preserving, and symbolic roles [22,23]. It is interesting to note that lavender does not play any meaningful role in ancient Roman cookery, at least not according to the recipes described by Apicius [24]. Nevertheless, evidence of its culinary use is not altogether absent from the historical record. For instance, Cunningham [25] observes that although violets and roses were used as edible flowers in Ancient Roman cuisine, lavender was also used to enhance the flavour of sauces. Laget [26] similarly highlights the difficulty of separating the culinary and medicinal histories of aromatic plants, questioning whether their early use was initially driven by their pleasant aroma or by perceived hygienic benefits. Against this broader historical background, Theophrastus (B.C. 371–287) listed the plant amongst Mediterranean flora, while Dioscorides (A.D. 40–90) described its medicinal properties and recommended its decoction for chest complaints [27]. Although lavender was one of the important medicinal plants of the ancient world, even Dioscorides [28] makes no mention of its culinary use. Historical documents additionally suggest that lavender became predominantly linked to bathing, fragrance production, domestic scenting, and therapeutic uses rather than culinary usage [8]. Curiously, an alternative path can be discerned in the case of saffron. Saffron, while esteemed in antiquity for various non-culinary applications such as cosmetics, perfumes, medicine, and textile dyeing, later gained significant prominence in the culinary realm as both a colouring and flavouring ingredient [29]. This disparity prompts a fascinating inquiry into why lavender, despite its equally enduring fragrant and therapeutic use, never experienced a similar shift toward a more prominent use in cuisine. Part of the answer may lie in what each plant offers the cook beyond its aroma. Saffron’s principal pigments, the crocins, are unusually water-soluble carotenoids with a very high colouring strength, so a few stigmas are sufficient to impart an intense golden-yellow colour to rice, breads, sauces, and sweets, while picrocrocin and safranal add a bitter-honeyed, hay-like flavour that is readily integrated into savoury as well as sweet foods [29,30]. Colour was highly prized in pre-modern cookery as a marker of luxury and status (yellow evoking gold), and saffron was one of the very few ingredients able to supply it [29,31]. Lavender offers no comparable functional property: its violet anthocyanin pigments are sensitive to heat and pH (as illustrated by the colour-unstable purple tea described for *L. stoechas* [32]), and they do not impart a strong or lasting colour to cooked food. Lavender’s only culinary contribution is therefore its aroma, and that aroma was already firmly anchored in bathing, perfumery, and linen. Saffron’s culinary transition may thus have been driven by a unique and hard-to-substitute property, namely colour, whereas lavender offered nothing that other, already-established culinary herbs could not supply. The Romans widely valued lavender for its aromatic, medicinal, and hygienic properties [27,33,34].

**Figure 2:**
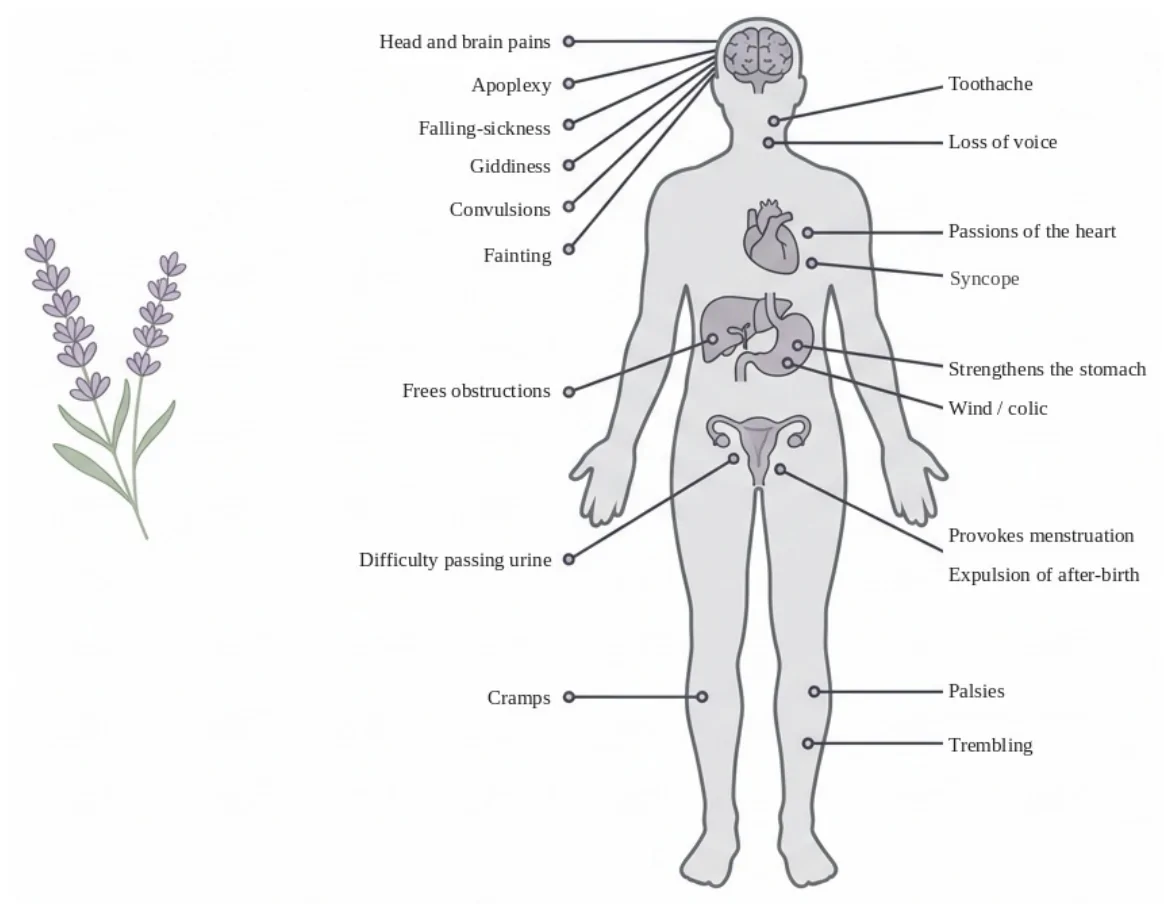
Traditional medicinal uses of lavender described by Culpeper [36]. The figure summarises the ailments and therapeutic applications attributed to lavender in *The Complete Herbal*, including uses relating to the head and brain, digestive and urinary systems, menstruation, toothache, fainting, palsies, and trembling. These uses reflect historical medical beliefs rather than contemporary clinical evidence. Adapted from [8,36].

Following antiquity, lavender’s standing evolved chiefly within medicinal, domestic, and fragrant domains. Mediaeval herbal texts and subsequent pharmacopoeias progressively documented lavender for its medicinal purposes, while evidence of its use as a culinary component is limited [8,35]. A notable example of lavender being regarded as beneficial for certain ailments is found in Culpeper [36], who advocated the herb’s use to alleviate “griefs and pains of the head and brain” resulting from a chilly origin, encompassing apoplexy, epilepsy, dropsy, cramps, convulsions, palsies, and fainting. Culpeper attributed to lavender the ability to fortify the stomach, alleviate blockages in the liver and spleen, stimulate menstruation, and remove a deceased foetus or the placenta. Lavender formulations were additionally advised for urinary retention, gas, colic, dizziness, and dental pain. Culpeper, however, warned that the concentrated “Oil of Spike” [37,38,39] was so potent and penetrating that merely a few drops should be applied for internal or external ailments (see Figure 2).

It should also be borne in mind that the separation of floral notes from savoury food is itself a relatively recent, and largely Western European, development. In mediaeval Arabic and Persian cookery, floral waters (above all rose water), flower-scented syrups, and aromatic blends combining flowers, spices, and resins were routinely used in both sweet and savoury dishes, as documented in the tenth-century Baghdadi cookbook of Ibn Sayyār al-Warrāq and the thirteenth-century *Kitāb al-Ṭabīkh* [40,41]. Much of this repertoire was transmitted to mediaeval European cookery, in which rose water, violets and other flowers, sugar, and spices were likewise combined in meat as well as in sweet dishes [31,42]. Only from the seventeenth century onwards, with the emergence of the new French cuisine, were sweet and savoury progressively separated into distinct courses and flavour registers, with sugar, many spices, and floral aromatics largely withdrawn from savoury cooking in Western Europe [42]. The scarcity of lavender in modern Northern European savoury cooking may therefore partly reflect this broader historical bifurcation of the Western palate, rather than anything specific to lavender in particular. That said, even in these floral-rich mediaeval traditions, lavender does not appear to have featured prominently, with rose, orange blossom, and violet being far more common, suggesting that the bifurcation provides, at most, a partial explanation.

### 1.3. Contemporary Use of Mediterranean Herbs

While Mediterranean herbs have long been used in the cuisine of the region, their use in Northern European cuisine has, until recently, been far less common [43]. In part, this may simply reflect the fact that while spices were traded over long distances, the use of herbs tended to be based on those plant varieties that were available locally [44,45].

For instance, according to commentators, it has been suggested that, following the Second World War, Mediterranean herbs [46] rarely appeared in the cuisine of the British Isles. According to certain commentators, the writings of Elizabeth David may have been responsible for the (re-)introduction of Mediterranean herbs into the British kitchen in the second half of the 20th Century [47]. However, it is worth noting that even she was sceptical about the culinary potential of rosemary and sage and at one point acerbically suggests that while such Mediterranean herbs make passable table decorations, they are too strong to incorporate in foods [48].

David [48] (p. 2) additionally presents historical instances demonstrating that lavender held a significantly distinct function compared to most culinary herbs. Instead of being commonly used in culinary practice, lavender and rosemary were scattered on domestic floors, while honey-infused beverages like mead, hydromel, and metheglin were perfumed with various fragrant plants, including lavender. These instances indicate that, while lavender was undoubtedly esteemed in English culinary traditions, its primary role was linked to aroma and the enhancement of drinks rather than conventional cooking [48] (p. 2).

There is certainly little mention of Mediterranean herbs in general, and lavender in particular, in Mrs. Beeton’s classic *Household Management* [49]. However, as has been noted already, even Italian cookbooks such as Apicius do not mention lavender [24,50]. Here, it might be objected that lavender is perhaps more strongly associated with France (and its cuisine) than with any other Mediterranean country. On this point, it is worth noting that lavender is sometimes added as a dried herb to *Herbes de Provence*, a classic French blend. That said, while widely popular in North America and international markets for its ability to provide a floral, sweet, and visually distinct profile, it is not a traditional ingredient in authentic French cooking or strictly certified blends [51].

According to Masters [32], topped lavender (*Lavandula stoechas*) is used to make a purple aromatic syrup in Turkey. This labour-intensive process involves removing the petals from the flowerhead by hand. Masters writes: “Flowerheads are also used whole to make tea. When made in regions where the water is slightly acidic, fresh flowerheads produce a purple-colored (but not color-stable) tea, while dried flowerheads lack the distinctive color but retain the flavor, which is slightly less floral than English lavender”. This is by no means an isolated example. In Türkiye, *L. stoechas* (known locally as *karabaş otu*) has a long tradition of use as a herbal tea [52], and the cultivation of *L. angustifolia* around Isparta has, in recent decades, given rise to a local lavender gastronomy that includes lavender-flavoured Turkish delight, ice cream, *muhallebi*, *gözleme*, and Turkish coffee [53,54,55]. In Morocco, too, *L. stoechas* is used to flavour teas, soups, and certain traditional dishes, including a particular type of couscous [56]. These regional traditions are important in the present context because the psychological evidence reviewed below, regarding lavender’s categorisation as a ‘non-food’ odour, comes almost exclusively from Northern European and North American participants. They suggest that lavender’s association with non-food uses is culturally contingent rather than universal and that, where lavender (often a different species with a different chemical profile;); is encountered in food and drink from an early age, it may well be categorised quite differently. Cross-cultural studies comparing the perceived edibility of lavender aroma in, for instance, Turkish and British consumers would provide a direct test of this suggestion.

An interesting reference from the eighteenth century is found in Arthur Murphy’s ‘What We Must All Come To’, in which a character depicts “a more exquisite sucking pig in lavender, with sage flourishing in its belly” [57] (p. 10). While the excerpt mentions a comical garden setup instead of a genuine meal, the depiction of sage flourishing in the pig’s belly can be taken to suggest the enduring gastronomic link between aromatic herbs such as sage and the preparation or flavouring of pork. Curiously, lavender is presented in a decorative or fragrant manner, while sage has a very culinary connotation.

Empirical research suggests that lavender aroma may not readily be perceived as food-related in the absence of a culinary context. For example, lavender has been classified with jasmine and lily-of-the-valley as a non-consumable scent, with the differentiation between consumable and non-consumable scents corroborated by initial evaluations of perceived edibility [58]. Hannum, Fryer, and Simons [59] similarly categorised lavender with honeysuckle, rose, and jasmine as a group of non-food tastes (presumably meaning flavours), distinguished by their relatively poor retronasal recognition. In alignment with this categorisation, non-food aromas seem to affect food-related conduct specifically due to them being recognised as separate from food. Kemps, Tiggemann, and Bettany [60] discovered that exposure to jasmine, categorised alongside lavender as a non-food fragrance, markedly diminished chocolate cravings compared to what they describe as a food scent (green apple) or a no-odour control. This finding indicates that lavender’s classification as a non-food aroma could potentially be used in a similar manner, despite limited investigation in this regard. It is, however, important to mention that these investigations were conducted in Northern Europe and North America, regions where the culinary application of the plant is somewhat uncommon. These results should obviously not be taken to suggest that lavender is fundamentally inedible; rather, they underscore the significance of familiarity, acquired connections, and culinary context in influencing the perception of an odour as food-related.

A further and perhaps more pervasive contributor to lavender’s perception as a non-food aroma may be the sheer ubiquity of lavender and other linalool-rich fragrances in non-food household products. As documented later (Section 4, Table 2), lavender features prominently in soaps, shower gels, shampoos, laundry detergents, dishwashing liquids, room and linen sprays, and furniture polish [61,62,63,64,65]. For many consumers in Northern Europe and North America, then, the most frequent encounters with lavender’s aroma occur in the bathroom, the laundry, and the bedroom, rather than in the kitchen or at the table. Through repeated co-occurrence, such odours may come to be categorised automatically as ‘cleaning’ or ‘personal care’ cues rather than as food cues [58,59]. When the same aroma is subsequently detected in food, it is likely to be matched to this non-food category, giving rise both to the familiar ‘soapy’ descriptor [66] and to the kind of conceptual incongruity outlined in the Introduction. In other words, lifelong exposure to lavender-scented cleaning and personal-care products may produce a form of crossmodal, category-based suppression of lavender’s perceived edibility, one that would be expected to be weaker in those regions where lavender is also encountered in traditional foods and drinks (see above). To the best of our knowledge, this testable hypothesis, which could be examined by relating individuals’ exposure to lavender-scented household products to the perceived edibility and liking of lavender-flavoured foods, has not yet been investigated directly.

In Northern Europe, one occasionally comes across lavender shortbread, and it is perhaps only here where its presence is perceptually apparent to the consumer. The title of an online article in *Bon Appétit* from June [66] put it as follows: “How to cook with lavender so your food doesn’t taste like soap.” The ‘soapy’ reputation of lavender’s scent seems to be significantly influenced by the specific cultivar and its quantity. Culinary lavender generally originates from low-camphor varieties of *Lavandula angustifolia*, while high-camphor Lavandin (*L.* × *intermedia*) hybrids or overuse may result in the soapy or fragrant flavour linked to lavender [67]. In the popular culinary literature, lavender is frequently mentioned in conjunction with rosemary, a similar herb from the same botanical family. Lavender is recognised for its compatibility with pastries and drinks, providing a delicate, sweet, and floral fragrance, while rosemary delivers a pungent, resinous flavour that enhances savoury cuisine [68]. In addition, lavender leaves can be steeped in milk or cream for ice cream, blended into syrups or sugar, or combined with herbs such as thyme, rosemary, bay leaf, and sage when roasting vegetables [69].

North American gins often incorporate lavender as a supposedly dominant note [32]. According to Masters [32], “Lavender shares aromatic properties with juniper due to the terpinen-4-ol they both contain. That means they pair well to create gin that is characteristically junipery, but with a little extra depth. Lavender brings the floral note of linalool in contrast to resinous pinene from juniper.” (Note, though, that spirits, e.g., aperitifs, often include ingredients that are by themselves very unpleasant, such as the bitter taste of wormwood; hence, perhaps lavender’s presence in gin should not be taken as relevant for the culinary use of this herb.)

## 2. Is There a Specific Volatile That Is Simply Incongruent with Cuisine?

Over 100 volatile compounds have been recognised in lavender, although the qualitative and quantitative makeup differ significantly based on genotype, geographical origin, climatic factors, cultivation techniques, harvest timing, and extraction methods [70,71,72]. Notwithstanding this chemical diversity, a limited number of constituents usually account for most of the oil, namely linalool, linalyl acetate, lavandulol, lavandulyl acetate, terpinen-4-ol, (*Z*)- and (*E*)-β-ocimene, 1,8-cineole, and camphor. Their relative proportions, however, are determined primarily by species, chemotype, and cultivar, and secondarily by growing conditions, harvest stage, and extraction method, rather than by the country of origin as such, and they vary so widely that lavender essential oil cannot meaningfully be reduced to a single, country-specific composition [70,72,73,74,75,76]. Table 1 therefore summarises the typical compositional profiles of the principal lavender taxa, together with their aroma character and culinary relevance. A recent comprehensive review confirms that linalool and linalyl acetate are the primary volatile constituents of lavender across all significant species, with linalool levels varying from less than 1% to more than 60% based on species, extraction technique, plant section, and cultivar [77]. A standardised lavender oil formulation has shown clinical effectiveness for anxiety in multiple randomised studies [77].

Linalool is a dominant volatile, responsible for lavender’s anxiolytic effects [84]. However, linalool is by no means unique to lavender and is also a major constituent of other culinary herbs and spices, including basil (*Ocimum basilicum*) and coriander (*Coriandrum sativum*) [85,86]. Perhaps the key question therefore relates not simply to the presence of linalool, but rather to its concentration and its interaction with other volatile constituents. Odour perception is rarely determined by individual molecules acting in isolation; instead, it emerges from complex mixtures in which changes in the concentration of a single compound may alter the overall perceptual quality disproportionately [87]. Moreover, the concentration of aroma compounds can obviously influence the perceived intensity/odour quality, with pleasant floral notes at low concentrations becoming medicinal, perfume-like, or even overpowering at higher levels [87,88]. Lavender’s significant connection to perfumery is pertinent here, as it is classified as a ‘head note’, generating an initial yet transient olfactory impression [69]. This may help to explain why lavender is generally appreciated when used sparingly in foods, whereas larger amounts are frequently described as ‘soapy’ or ‘perfumed’, despite containing volatile compounds (such as linalool) that are common to many accepted culinary herbs.

Terpinen-4-ol represents another major lavender volatile. Although commonly associated with lavender, it is also a characteristic constituent of juniper berries, tea tree, marjoram and other aromatic plants, and contributes to the characteristic aroma of gin through its presence in juniper [89,90]. More generally, it remains unclear whether the reported effects are attributable to the volatile compound itself or to the perceptual attributes that are associated with it. The occurrence of 1,8-cineole and linalool is therefore not exclusive to lavender, even within what might be described as Mediterranean culinary herbs. For example, the essential oil of bay leaf (*Laurus nobilis*) contains 1,8-cineole (31.9%), along with sabinene and linalool, and exhibits antibacterial and antifungal properties [91], suggesting that these biological effects are characteristic of aromatic herbs more broadly, rather than being specific to lavender. This situation raises the possibility that some of the responses that are commonly attributed to lavender may also depend on how the plant’s aroma is perceived and interpreted.

Placebo and expectancy effects may also contribute to responses associated with lavender aroma [92]. An expanding body of research indicates that perceptions of a scent can significantly affect both personal and physiological reactions. In a double-blind placebo-controlled trial, Howard and Hughes [93] revealed that the participants’ expectations, rather than the presence of the lavender scent itself, predominantly influenced variations in psychophysiological relaxation after experiencing stress. Similarly, Masaoka et al. [94] found that verbal information regarding the analgesic properties of lavender significantly enhanced pain relief, indicating that expectancy contributed independently of the odour itself. It is important to recognise that anticipation might influence the perceived effects of exposure to lavender. Higuchi, Shoji, and Hatayama [95] discovered that individuals informed beforehand about lavender’s relaxing properties experienced a markedly greater reduction in stress after inhaling it compared to those who received no such prior information, indicating that a portion of lavender’s alleged soothing effect may stem from placebo-like expectations rather than solely its chemical composition. Contextual information and verbal descriptors can profoundly influence the perception, interpretation, and assessment of a scent’s edibility [96,97]. Given these discoveries, the inquiry into whether lavender is treated differently when showcased in a distinctly culinary setting is significant. For example, lavender used in a shortbread recipe may be classified as a flavouring agent instead of predominantly a fragrance; this could diminish the expectation-driven effects linked to lavender. Nonetheless, this potential has not been subjected to empirical examination as yet.

## 3. Does Lavender’s Anxiolytic Effects Preclude Its Use in the Kitchen?

It is worth noting that one finds mention of the strewing of lavender [98], and its association with bed/bedsheets, stretching back more than 600 years [99]. While some have questioned its anxiolytic (relaxing) properties [100], evidence from Harada et al. [84] shows effects in mice, specifically related to olfactory inhalation (though one may still question whether the concentration presented to mice is realistic for human use). Over the years, there have been many reports of lavender being used to help relax people [101,102]. (It is noticeable that the beneficial effects are often demonstrated in what might be defined as a need state.) [103].

### 3.1. Anxiolytic Effects of Lavender: Aromatherapy and Oral Administration

A systematic review and meta-analysis of 22 randomised controlled trials with more than 2100 participants revealed that lavender aromatherapy resulted in a moderate-to-large decrease in self-reported anxiety (Hedges’ ĝ = −0.65), as well as notable reductions in systolic blood pressure, heart rate, and salivary cortisol, with inhalation yielding somewhat greater effects than massage or oral (silexan) administration [104]. Relevant here, a systematic review and meta-analysis conducted by Ghavami, Kazeminia, and Rajati [105] demonstrated a robust effect of lavender on people’s self-reported stress, implying that, for whatever reason, the smell of lavender ‘does what it says on the tin’! In total, 21 studies including data from 1595 participants were included in the meta-analysis. In addition to studies involving adults, lavender aromatherapy has demonstrated quantifiable advantages in children: a crossover randomised trial by Ghaderi and Solhjou [106] revealed that ambient lavender diffusion during dental restorative procedures significantly lowered salivary cortisol, heart rate, and self-reported pain in 7–9-year-olds compared to a control session without aroma.

Inhaled lavender essential oil did not significantly reduce mental stress as compared with placebo in a randomised trial involving patients who were recovering from coronary artery bypass surgery [107]. Most vital signs also showed no significant improvement, although a small reduction in systolic blood pressure was observed. These findings suggest that lavender inhalation does not necessarily alleviate stress in postoperative patients. Evidence for the anxiety-reducing effects of orally administered lavender oil should be distinguished from evidence concerning its smell. Kang et al. [104] included oral silexan in their analysis, meaning that their findings are not confined to inhalation. Silexan, a standardised oral formulation of lavender oil, possesses significant clinical backing [14]. In generalised anxiety disorder, dosages of 80–160 mg/day surpassed placebo and demonstrated effectiveness similar to paroxetine, while resulting in fewer side effects [108]. A meta-analysis comprising five trials (n = 1213) reported benefits for anxiety and quality of life, with an effect size of 0.35 and a number-needed-to-treat of 5 [109,110,111]. These findings concern a standardised oral preparation and do not establish whether the use of lavender in food would produce comparable effects.

Some aromatherapy studies have reported reduced alertness, memory, and cognitive performance alongside reductions in anxiety [14]. Proposed mechanisms for lavender’s effects include serotonergic and glutamatergic pathways [14,112]. However, neither of these proposed mechanisms, nor findings from inhalation studies, establish that consuming lavender in food impairs cognitive performance. Oral silexan did not impair driving performance after single or repeated doses [113], and clinical trials indicate no significant sedation or withdrawal symptoms, with adverse effects predominantly limited to mild gastrointestinal complaints [109].

The effects of concentrated lavender oil preparations on mood cannot be assumed to apply to the amounts of lavender flowers used to flavour foods and drinks. To put these amounts into perspective, pharmacopoeial-grade dried lavender flowers must contain at least 13 mL/kg (approximately 1.3%) of essential oil [114], and contents of approximately 1–3% are typical [78]. A shortbread recipe using 1–2 g of dried flowers for a batch of 16–24 biscuits would therefore contain, before any losses during baking, roughly 13–60 mg of essential oil in total, or approximately 0.5–4 mg per biscuit. This is one to two orders of magnitude less than a single 80 mg capsule of Silexan, and it will be reduced further by volatilisation during baking (Section 4). Even the lavender tea discussed below, prepared from 2 g of flowers twice daily, corresponds to the upper end of the traditional posology (i.e., the dose, frequency, route and duration of administration) for lavender flower infusions (1–2 g in 150 mL of water, three times daily) [115], rather than to typical culinary use. One trial examined lavender consumed as tea: older adults who drank tea prepared with 2 g of dried lavender twice daily for two weeks reported lower anxiety and depression than an untreated control group, with no adverse effects reported [112]. Although this study did not establish whether lavender affects alertness or cognitive performance, or whether its findings extend to other culinary uses, neither does it provide any direct support for avoiding lavender in food because of presumed sedation. Lavender’s limited gastronomic acceptance may instead partly reflect its cultural association with relaxation rather than appetite, as discussed below. In summary, the clinical literature on concentrated oral and inhaled preparations cannot be extrapolated to culinary use, and there is no evidence, nor any pharmacological reason to expect, that lavender used at flavouring levels induces sedation or cognitive impairment, particularly given that even the far higher oral doses of Silexan did not impair driving performance [113].

### 3.2. Other Ambient Uses of Lavender Odour

In addition to its medical and gastronomic significance, the soothing fragrance of lavender is frequently used in purely atmospheric, non-consumable settings—such as scents used in buildings and workplaces—where the perfume alone, rather than any consumed substance, is believed to be effective. A significant portion of this ambient effect seems to depend on a singular volatile compound, linalool, which elicits similar relaxing effects even when derived from completely different plant sources. Kuroda et al. [116] discovered that the aroma of jasmine tea and (R)-(−)-linalool resulted in quantifiable sedative effects on autonomic nerve activity and self-reported mood states, closely mirroring the pattern observed for lavender’s linalool-dominant profile (refer to Section 2). This compound-level convergence implies that lavender’s widespread association with relaxation may be attributed as much to a common chemical profile among otherwise disparate aromatic flora as to any unique characteristic of lavender itself.

Spence [103] reviewed the literature concerning the use of ambient fragrance to improve well-being, highlighting substantial evidence for the positive impact of ambient lavender aroma on sleep quality throughout many stages of life, including domestic, hospitality, and care environments. Nonetheless, these outcomes can by no means be assured: with over 45 species and 450 cultivated variations of lavender available, few investigations clarify which specific kind was used, and the findings might be significantly influenced by context. In a particular office study, the presence of ambient lavender fragrance paired with nature-sound music yielded no significant benefits compared to a silent, unscented control, highlighting that the impact of a scent relies on its harmonious integration with the environment rather than the scent itself [117,118]. This concept is effectively exemplified by the residential structure “House in Travessa do Patrocinio” [119], where aromas are intentionally allocated to correspond to the purpose of each level: rosemary, a more stimulating and pungent herb, adorns the exterior facade of the ground floor designated for communal spaces, lavender is assigned to the comfortable second-floor sleeping quarters, and saffron fragrances the rooftop swimming pool. Significantly, within this architectural exhibition, lavender is designated for the area primarily linked to relaxation instead of any communal or dynamic environment, thereby affirming its entrenched cultural symbolism as a symbol of tranquillity (cf. Section 3, above). Meanwhile, rosemary, rather than lavender, adorns the structure’s most visible, socially engaging section.

**Figure 3:**
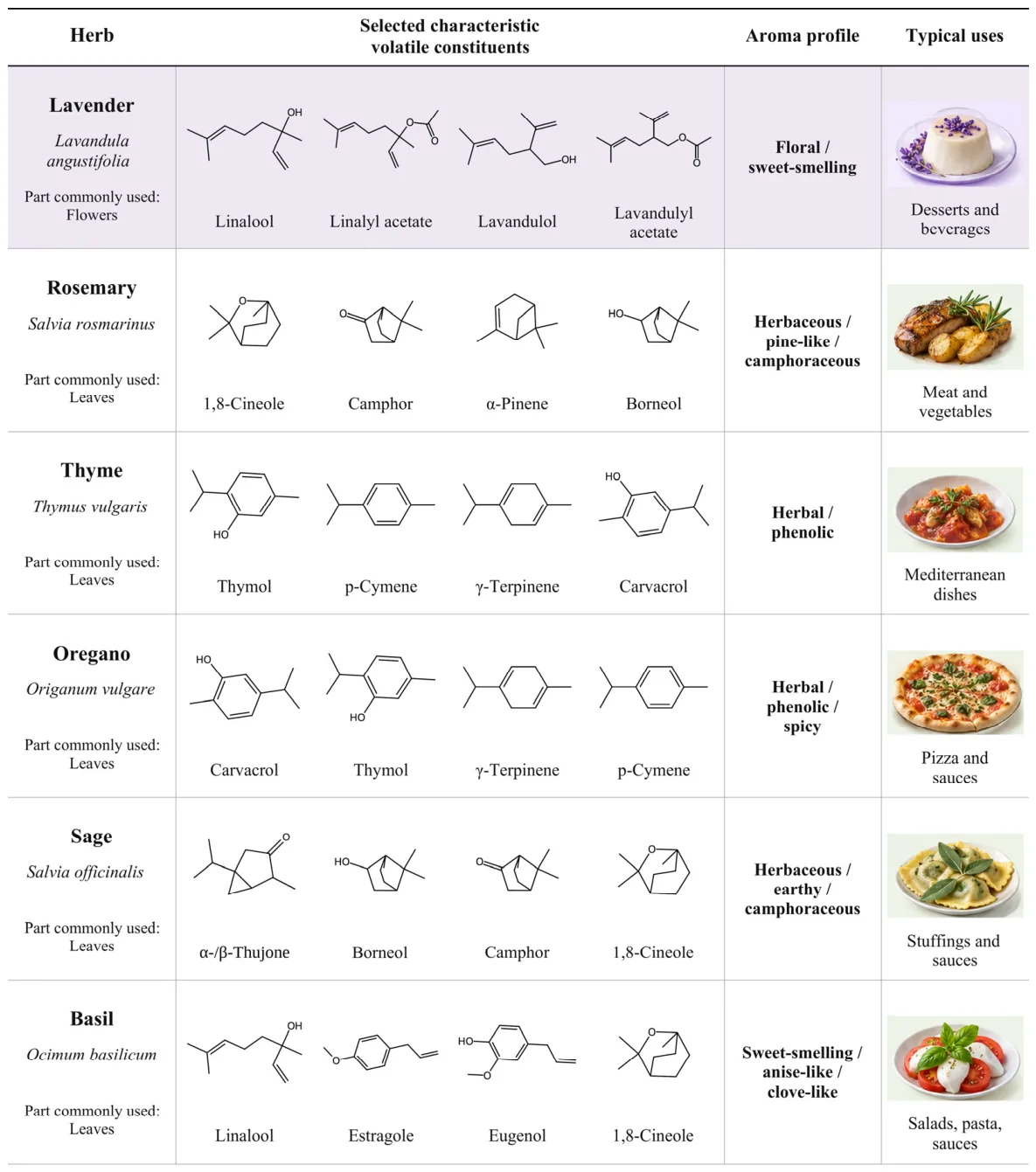
Parts commonly used, selected characteristic volatile constituents, aroma profile, and typical culinary uses of lavender and selected Mediterranean herbs. Key constituents shown: lavender (*L. angustifolia*), linalool, linalyl acetate, lavandulol, and lavandulyl acetate; rosemary, 1,8-cineole, camphor, α-pinene, and borneol; thyme, thymol, *p*-cymene, γ-terpinene, and carvacrol; oregano, carvacrol, thymol, γ-terpinene, and *p*-cymene; sage, α-thujone, β-thujone, camphor, and 1,8-cineole; and basil (European type), linalool, estragole (methyl chavicol), eugenol, and 1,8-cineole [75,86,127,134,135]. The relative abundance of these compounds varies with chemotype and cultivar (Table 1). Chemical structures were generated from their standard structural identifiers using the RDKit cheminformatics toolkit; the dish images illustrating typical culinary uses are AI-generated illustrations and are provided for illustrative purposes only.

The scent of lavender seems to serve as a contextual signal for memory, reflecting results observed with other Mediterranean herbs. For instance, Ball, Shoker, and Miles [120] revealed that the presence of ambient rosemary scent during encoding and subsequently reintroduced at retrieval resulted in significant context-reinstatement effects on indirect measures of memory, despite participants frequently being oblivious to the scent’s impact. Considering that lavender contains linalool and various other volatile compounds similar to rosemary (Table 1; Figure 3), it is conceivable that a similar context-dependent memory effect for lavender scent exists; however, to our knowledge, it has not been empirically examined. This aligns with lavender’s long-standing connection to bedrooms and bedding, since a consistently soothing aroma associated with relaxation and slumber may, via frequent exposure, serve as a conditioned stimulus for that state, irrespective of any medicinal effects.

### 3.3. Lavender and Sleep

Lavender’s effects on sleep may vary depending on the research population’s characteristics, the method of distribution, and how pleasant the scent is experienced as being. A randomised trial was carried out by Li et al. [121] with the express purpose of comparing delivery techniques and odours for use at night. In comparison to no smell stimulation, they discovered that a diffused aroma enhanced subjective sleep quality and decreased weariness the following day. Lavender by itself, however, did not provide any benefit above the untreated control condition. Rather than lavender specifically, the effects were primarily driven by the distribution mechanism and overall pleasantness of the smell. Furthermore, if nightly exposure was discontinued, no advantage remained. There were no discernible variations in objective, wearable-tracked sleep metrics. When viewed in the context of the larger literature on aromatherapy, this trend aligns with a finding regarding the psychological advantages of pleasant scents. When a person is already in a “need state”—that is, anxious, agitated, or sleep-deprived—these benefits are more consistently shown. In a healthy, well-rested population with minimal opportunity for improvement, they are, perhaps unsurprisingly, less consistently shown [92,122,123,124]. Therefore, at least as much as any deficit in the molecule itself, lavender’s apparent unreliability as a sleep aid in some trials may indicate a mismatch between the research population and outcome measure. Every time lavender’s psychoactive claims are examined under various circumstances throughout this review, this pattern appears (see Section 3, above). Collectively, these insights suggest that the calming and sleep-inducing properties of lavender are significantly influenced by environment and cannot be universally used for culinary uses. This has special importance when contrasting lavender with other Mediterranean herbs, as its growing culinary use demands comparisons with herbs that possess a deeper entrenched tradition in culinary settings.

### 3.4. Culinary Contributions of Mediterranean Herbs

The role of lavender in the kitchen becomes clear when compared to other fragrant herbs having a Mediterranean origin. Numerous entities are already well entrenched in both regional and worldwide cuisines. Other herbs from the Mediterranean, including sage, have demonstrated antioxidant characteristics [125]. Recent chemical investigations have identified shared antioxidant compounds in these herbs, while also showing differences in their overall antioxidant potential. Płatkiewicz et al. [126] discovered that oregano, sage, basil, and rosemary possess a shared hydroxycinnamate-derived phytochemical framework, predominantly consisting of rosmarinic acid. However, they vary significantly in total antioxidant potential, with oregano regularly surpassing the others in multiple assay techniques. A comparative analysis of oregano and thyme arrived at a comparable outcome with more specificity. The two herbs are characterised by the presence of identical monoterpenes, thymol and carvacrol. Thyme possesses significantly greater levels of rosmarinic acid, although oregano excels in overall phenolic content [127]. Collectively, these examinations suggest that “Mediterranean herb” does not represent a singular flavour or chemical composition, but rather a collection of interconnected, partially overlapping chemical profiles. This discovery accurately corresponds with the distinct species chemotype variation seen in lavender (refer to Section 2).

Mediterranean herbs can stimulate the trigeminal system, contributing sensations such as warmth, cooling, or tingling to the overall flavour experience [128,129]. Xu, Delling, Jun, and Clapham [130] claim that oregano and savoury, another herb, stimulate particular TRP channels through their characteristic chemicals, carvacrol and thymol [131,132]. A later analysis conducted by Rhyu, Kim, and Lyall [133] contextualises this discovery within the wider domain of chemesthesis (chemical feeling). The activation of TRPA1 and TRPV1 receptors by these substances does not solely induce warmth or discomfort. It also affects taste perception, altering the perceived strength of bitter, sour, and salty tastes via common brain pathways [129,133]. By contrast, chemesthesis appears to contribute little to the flavour profile of lavender. Lavender is predominantly experienced through olfactory senses rather than trigeminal nerve activation (Section 2). This is a moment when lavender’s culinary essence separates from its more aromatic Mediterranean relatives. This difference has practical consequences in the kitchen. In savoury cooking, much of the ‘backbone’ contributed by thyme, oregano, and savoury, and to a lesser extent by rosemary and sage, derives from the oral warmth and mild pungency produced by phenolic monoterpenes such as thymol and carvacrol acting on TRPA1 and TRPV1, and from the cooling or warming sensations elicited by 1,8-cineole and camphor [128,129,130,131,132,133]. Lavender’s dominant constituents, linalool and linalyl acetate, are comparatively weak chemesthetic stimuli at culinary concentrations, so lavender contributes an aroma with little oral ‘bite’ to anchor it within a savoury dish. On its own, it therefore tends to be perceived as a perfume sitting on top of the food, rather than as a seasoning integrated into it. This may help to explain why lavender’s most successful savoury and non-sweet applications are those in which it is combined with pungent leaf herbs (as in *Herbes de Provence*, alongside thyme, rosemary, and savoury) or with resinous botanicals (as with juniper in gin; Section 1.3), which supply the trigeminal and resinous structure that lavender itself lacks [32,51] (see Figure 3).

Lavender differs from many Mediterranean herbs in the part of the plant used for cooking. It is important to highlight that the lavender flower is used, in contrast to the leaves of other Mediterranean herbs that are typically ingested [136]; in fact, the previously mentioned comparator herbs—sage, oregano, thyme, rosemary, and basil—are recognised and referenced in the literature as leaf herbs instead of flower herbs [137,138]. The concentration and composition of essential oils can vary significantly among different plant organs, even within the same species [139,140], indicating that this botanical differentiation is not just a descriptive feature. It may elucidate why lavender holds a relatively minor culinary role compared to herbs whose edible portions are predominantly leaves.

## 4. Is Supply/Preservation/Drying Any Different for Lavender Compared to Other Mediterranean Herbs?

Before considering whether supply or preservation might constrain lavender’s culinary use, it is useful to consider the remarkable breadth of products in which lavender is already incorporated. Table 2 shows how modern uses go far beyond food and drink to include cosmetics, domestic goods, aromatherapy, biomedical applications, pest-control formulations, technical textiles, packaging materials, and even products made from waste from lavender processing. This wide range of uses implies that lavender is neither economically marginal nor intrinsically challenging to use in manufactured goods.

Lavender and its derivatives have been integrated into a variety of products with significantly diverse matrices and processing demands, including food and beverages, wound dressings made from nanoscale fibres, active packaging, functional textiles, and materials derived from post-distillation biomass [145,147,169,170,175,176,181,183]. Thus, the pertinent question is not why lavender is often underused, but rather why its widespread application in other areas has not resulted in similarly widespread everyday use in the kitchen.

Beyond benefiting from its culinary and health-related potential, lavender possesses substantial economic significance. Bulgaria and France together generate about two-thirds of the global essential oil production from lavender, with the overall supply growing in recent years to meet the escalating demand from the fragrance, cosmetics, and, increasingly, food industries [184]. In short, the answer is no: that is, there would not seem to be any supply/pricing issues that might explain why it is that lavender is not commonly used as a Mediterranean culinary herb [185]. The aromatherapeutic use of this particular Mediterranean herb does not lead to higher prices, or reduced availability, of this herb in the kitchen setting.

Drying and extraction conditions can alter lavender’s chemical composition and aroma: Radu et al. [186] found that the thermal treatment of lavender extract (75–100 °C) significantly altered its phytochemical profile, particularly chlorophylls and carotenoids, in a manner contingent upon temperature and duration, while total flavonoids remained predominantly stable and total polyphenols exhibited slow degradation across the studied spectrum—a pattern of heat sensitivity prevalent among various other dried culinary herbs, rather than one that is distinctive to lavender. Danh et al. [187] further exemplify the significance of processing conditions by comparing hydrodistillation, hexane extraction, and supercritical CO_2_ extraction. Hydrodistillation conducted at 100 °C produced the highest linalool concentration but the minimum linalyl acetate, aligning with the partial thermal degradation of the ester, whereas the lower-temperature supercritical CO_2_ extract showed negligible thermal degradation and had the aroma that most closely resembled, although it was not identical to, that of the starting plant material. A corresponding pattern has been recorded in other desiccated Lamiaceae plants. Sensory evaluation of rosemary (*Rosmarinus officinalis*) indicated that oven-drying altered its distinctive ‘fresh’, ‘pine’, and ‘herbaceous’ attributes to ‘hay-like’, ‘spice’, and ‘woody’ characteristics, with commercial samples aged for six months exhibiting the most significant transformations [188]. This implies that the loss of flavour due to drying is a common characteristic of culinary herbs, not just exclusive to lavender.

The same thermal sensitivity is directly relevant to culinary preparation. Linalool (boiling point c. 198 °C) and linalyl acetate (c. 220 °C) are sufficiently volatile to be lost rapidly by evaporation and steam distillation from the surface of doughs, batters, and simmering liquids at normal cooking temperatures [189]. Moreover, with heating, particularly in aqueous or mildly acidic media, linalyl acetate is hydrolysed to linalool, while linalool can itself undergo rearrangement and oxidation to compounds such as α-terpineol, geraniol, nerol, and the linalool oxides, whose odour qualities are more woody, earthy, or ‘soapy’ than the fresh, fruity–floral note of the intact ester [10,187,189]. Because the lightest, freshest ‘top’ notes are lost preferentially [69], prolonged baking, boiling, or steeping will tend to shift the balance of what remains towards the less volatile camphoraceous and resinous constituents (camphor and borneol, particularly in lavandin-rich material; Table 1) and towards non-volatile phenolic compounds extracted from the flowers and calyces, such as rosmarinic acid and other hydroxycinnamic acids, which can contribute bitterness and astringency [78,186]. This may help to explain why raw or gently infused lavender (in syrups, sugars, creams infused below boiling point, or used as a finishing garnish) smells pleasantly floral, whereas heavily baked, boiled, or over-steeped applications are more often described as bitter, resinous, or soapy [66,67]. It is also consistent with common culinary advice to infuse lavender into warm, rather than boiling, liquids for short periods, to strain out the flowers, or to add lavender towards the end of cooking. Systematic sensory studies comparing lavender flavour before and after typical baking and cooking regimes are, however, currently lacking.

The food matrix also influences how lavender is perceived. Linalool and linalyl acetate are lipophilic (log *P* of approximately 3 and 4, respectively), so that in high-fat matrices, such as butter, cream, and chocolate, a large proportion partitions into the lipid phase, lowering the headspace concentration and slowing their release during eating [190,191]. In aqueous systems, such as teas, syrups, and lemonades, by contrast, the same amount of lavender produces a higher air-to-product partition and hence a more immediate, intense, and perfume-like aroma. Fat therefore acts as a buffer that effectively raises the detection threshold and flattens the release profile, which may partly explain why lavender is more readily accepted in shortbread, ice cream, and chocolate than in lean or aqueous preparations, and why the latter require considerably lower doses. These matrices are, moreover, typically sweet, and the repeated pairing of floral odours with sweet tastes can lead such odours to be perceived as ‘sweet-smelling’ and congruent with desserts [192]. Both mechanisms point towards dessert and confectionery applications as the most forgiving contexts for lavender, a prediction that is consistent with the food products documented in Table 2.

## 5. Chemical and Sensory Complexity of Lavender: The Role of Cultivar Variation

In fact, like for many other herbs, there are actually a large number of varieties of lavender, each presenting a somewhat different composition of volatiles [193]. For instance, if one takes the four cultivars Buena, Munstead, Royal Velvet, and Provence, the first three are selections of English lavender, whereas Provence is a hybrid of English lavender and the more pungent spike lavender (*Lavandula latifolia*). Hidcote, another cultivar of English lavender, is also worth seeking out for its deep blue-violet flowers, which retain colour as well as aroma when dried. Some cooks, by contrast, prefer French lavender (*L. stoechas*), whose more resinous, camphor-rich aroma suits robust savoury dishes better than sweets. This kind of cultivar-level variation is not merely anecdotal: Barut Gök and Erdoğdu [79], for instance, showed that different lavender cultivars grown under the same conditions can diverge markedly in their essential oil yield and volatile profile, underlining just how much “lavender” as a flavour ingredient depends on which variety is actually being used. From a practical standpoint, Table 1 suggests that food formulators should favour low-camphor *L. angustifolia* cultivars; that lavandins (including ‘Provence’, which is a hybrid of English and spike lavender) and spike lavender, with their higher camphor and 1,8-cineole contents, are better suited to perfumery and household products; and that *L. stoechas*, dominated by fenchone and camphor, is best reserved for the regional teas and syrups in which it has a traditional role.

Volatile compounds contribute to lavender’s characteristic aroma. The correlation between the molecular structure and fragrance of linalool, linalyl acetate, and their corresponding oxygenated derivatives suggests that little modifications in molecular arrangement can greatly influence the perceived aroma [10]. By contrast with oregano and thyme, whose phenolic monoterpenes activate particular TRP channels [130], lavender’s principal constituents contribute little chemesthetic stimulation, so its sensory impact is predominantly olfactory (Section 3.4).

Some herbs, such as oregano and thyme, stimulate particular TRP channels involved in chemesthesis—the perception of sensations such as burning, cooling, and/or tingling [130]. The flavour of lavender has always been associated, albeit informally, with its antibacterial and therapeutic properties [194], yet this relationship is not as simple as it appears. The straightforward anti-microbial concept, which was subsequently invalidated as a comprehensive explanation: Bromham et al. [195] discovered that the historical link between a plant’s application as a spice or flavouring and its antibacterial properties is significantly less robust and more variable than previously suggested. Many other substances possessing therapeutic effects are present in food, indicating that flavour and antibacterial efficacy may essentially be independent attributes that coincidentally coexist rather than one simply elucidating the other.

According to Prusinowska and Śmigielski [78], lavender essential oil has good antioxidant and antimicrobial activities and a significant positive effect on the digestive and nervous systems [36,196,197]. Aguerd et al. [198] similarly report significant antioxidant and antibacterial properties of lavender essential oil, emphasising that, regardless of the exact correlation between flavour and antimicrobial efficacy, the biological activity of lavender is firmly substantiated. The culinary significance of these discoveries, therefore, hinges on whether similar effects manifest at the concentrations and forms of lavender commonly used in cuisine.

## 6. Conclusions

Taken together, therefore, it would appear that lavender’s physiological/psychological relaxing effect (and hence its cognitive association), rather than anything intrinsic about the volatile compounds that make up its aroma/flavour, may provide the most plausible explanation for why this Mediterranean herb is not used more frequently in cuisine. The suggestion is that lavender’s association with a scenting/calming function for centuries may discourage this flavour, when distinctive, from being happily incorporated in our food. As such, there would appear to be limited grounds for recommending the renewed culinary use of lavender without considering how its distinctive flavour is incorporated. One approach is to use it in a form that masks or blends its distinctive character, as is the case in *Herbes de Provence* and in North American-style gins. From a gastronomic perspective, these instances imply that the acceptance of lavender in cuisine may be dependent upon the setting in which its flavour is experienced. Its integration into well-known dishes and beverages may enable its flower essence to enhance without overshadowing the overall sensory perception. The choice of cultivar, amount, and method of preparation are pertinent in this context, especially considering the distinctions between low-camphor culinary lavender and more aromatic types. These reflections indicate that there could be potential for broadening its gastronomic application via meticulous formulation and combination. The present review points to several practical levers in this regard: choosing low-camphor *L. angustifolia* (Table 1); limiting heat exposure by infusing gently or adding lavender late in preparation; favouring fat-rich and sweet matrices that buffer its release; and pairing it with pungent herbs or resinous botanicals that supply the trigeminal structure it lacks. Finally, the lavender-rich culinary traditions of Türkiye and Morocco, and the pervasive use of lavender in household cleaning and personal-care products elsewhere, suggest that lavender’s ‘non-food’ status is learned and culturally contingent, rather than intrinsic to the plant.

Lavender’s potential health benefits provide an additional reason to explore its use in food and beverages. The anxiolytic, antioxidant, and antibacterial results discussed previously may be pertinent to the creation of food and beverages that leverage its connection to calm. Nonetheless, data acquired from essential oils or standardised formulations do not demonstrate the same advantages as when used at much lower concentrations in most culinary applications. The allure of lavender as a wellness-related component must be differentiated from proven health benefits in the final meal or beverage. Subsequent investigations may examine if the acceptance of a lavender-flavoured item, which is otherwise comparable, varies when categorised as a dessert, a savoury condiment, or a health-focused product. Such comparisons would assist in determining if lavender’s enduring link to tranquillity and slumber serves as an impediment to its culinary appeal or provides a benefit in specific gastronomic context. Lavender’s culinary future may depend as much on the expectations it evokes as on the flavour it delivers.

## Figures and Tables

**Table 1:** Comparison of the main lavender taxa: typical composition of the essential oil, aroma character, and culinary relevance.

Taxon (Common Name)	Characteristic Major Constituents (% of Essential Oil)	Camphor; 1,8-Cineole	Aroma Character	Culinary Relevance
* L. angustifolia * Mill. (true or English lavender; cultivars such as ‘Hidcote’, ‘Munstead’, ‘Maillette’)	Linalool c. 25–45; linalyl acetate c. 25–45; lavandulyl acetate c. 1–8; terpinen-4-ol c. 0.1–8; (*Z*)-/(*E*)-β-ocimene c. 1–10 [70,75,76,78,79,80]	Both low (camphor typically <1; 1,8-cineole typically <3)	Sweet, floral, fruity-fresh	Preferred culinary lavender (low camphor): desserts, baked goods, dairy products, chocolate, syrups, teas; best used sparingly [67]
* L. * × *intermedia* Emeric ex Loisel. (lavandin; clones such as ‘Grosso’, ‘Abrial’, ‘Super’, ‘Provence’)	Linalool c. 25–45; linalyl acetate c. 15–40; borneol c. 1–4 [76,81,82]	Both moderate (camphor c. 4–12; 1,8-cineole c. 3–12)	Floral but sharper, herbaceous, camphoraceous; ‘soapy’ at higher doses	Mainly perfumery, soaps, and detergents; limited culinary use (some lower-camphor clones, such as ‘Provence’, are sold for culinary use, at low doses) [67]
* L. latifolia * Medik. (spike lavender)	Linalool c. 30–50; linalyl acetate usually <2 [38,39,75,83]	Both high (camphor c. 8–20; 1,8-cineole c. 15–40)	Camphoraceous, eucalyptus-like, medicinal	Unsuitable as a food flavouring; historically used medicinally (Culpeper’s ‘Oil of Spike’) [36]
* L. stoechas * L. (topped, French or Spanish lavender)	Fenchone c. 15–75; linalool and linalyl acetate usually minor [52,56,75]	Camphor variable, often high (c. 3–55); 1,8-cineole c. <1–8	Camphoraceous, minty-resinous, less floral	Regional traditional uses: syrups and teas (Türkiye), teas, soups, and couscous (Morocco) [32,56]

Values are approximate ranges (% of total essential oil) compiled from the cited studies. They vary widely with chemotype, cultivar or clone, plant part, harvest stage, and extraction method, and should be regarded as indicative rather than definitive. Commercial oils are specified by ISO 3515 (lavender), ISO 8902 (lavandin), and ISO 4719 (spike lavender) [80,82,83].

**Table 2:** Documented contemporary products and applications of lavender and lavender-derived materials.

Use Area	Subcategory	Documented Product/Form	Source
Basic raw materials and intermediate products	Dried flowers and buds	Dried lavender for culinary use or for making scented sachets	[61,141]
	Essential oil	Lavender oil obtained by distillation	[142]
	Hydrosol/floral water	Lavender hydrosol; also sold as a formulation ingredient	[143]
	Culinary extract	Lavender extract added to foods and beverages	[144]
	Concentrated botanical extract	Extracts obtained using ultrasound and supercritical CO_2_ methods	[145]
	Microencapsulated powder	Powder form in which lavender extract is encapsulated in carrier materials	
Food products	Cookies and biscuits	Biscuits containing lavender flower powder; cocoa–lavender cookies	[146]
	Bread	Bread supplemented with lavender-processing residue	[147]
	Cakes and pastries	Lavender-flavoured cakes and pastries	[53]
	Ready-to-bake mixes	Lavender scone, brownie and shortbread mixes	[148,149]
	Macarons/”macaroons”	Product enriched with lavender extract; the publication uses the term “macaroons”	[145]
	Gözleme	Lavender-flavoured gözleme (a traditional Turkish thin flatbread, rolled out by hand, filled and cooked on a griddle)	[53]
	Ice cream	Ice cream containing lavender essential oil or encapsulated extract	[145,150]
	Yoghurt	Yoghurt supplemented with lavender powder	[151]
	Cheese	Fresh goat cheese containing lavender and fennel pollen	[152]
	Milk-based dessert	Lavender-flavoured pudding (muhallebi)	[53]
	Chocolate	Lavender chocolate	[61]
	Confectionery	Lavender lollies and candies	[153]
	Turkish delight	Turkish delight made with lavender hydrosol; pomegranate–lavender Turkish delight	[54,55]
	Jams, jellies and marmalades	Lavender jams; fruit–lavender combinations; lavender jelly and marmalade	[148,153]
	Mustard	Lavender mustard	[153]
	Chutney	Lavender chutney	
	Sweet sauces	Lavender chocolate sauce; rum, raisin and lavender chocolate sauce	[144,153]
	Flavoured sugar	Lavender sugar; vanilla–lavender and lemon–lavender sugar	[144]
	Flavoured salt	Lavender sea salt and Himalayan salt; herb-blended salt	[141]
	Spice and seasoning blends	Lavender pepper, Herbes de Provence containing lavender, and meat/poultry/fish seasonings	
	Flavoured oil	Olive oil infused with lavender and herbs; salad oil	
	Flavoured vinegar	Lavender red-wine vinegar, balsamic vinegar and white balsamic vinegar	[141,154]
	Lavender-infused honey	Floral honey infused with lavender	[148]
	Lavender-nectar honey	Honey produced from nectar collected by bees from lavender flowers	[155]
	Soup	Lavender-flavoured soup	[53]
Beverages and beverage ingredients	Lavender tea	Herbal tea/infusion containing lavender	[112,153]
	Tea blends	Lavender–chamomile, lavender–mint and lavender–Earl Grey	[141]
	Coffee	Ground coffee with lavender; lavender-flavoured Turkish coffee	[53,148]
	Lemonade	Lavender or honey–lavender lemonade	[53,155]
	Syrups and beverage concentrates	Lavender syrup; raspberry–lavender and vanilla–lavender syrups	[144]
	Kombucha	Fermented tea containing lavender flowers	[156]
	Beer	Wheat beer brewed with lavender flowers and honey	[157]
	Gin	Gin using lavender as a botanical ingredient	[158]
	Liqueur	Lavender liqueur	[159]
	Cocktail flavourings	Lavender bitters; extracts intended for cocktail use	[149,158]
Cosmetics and personal care products	Bar soap	Lavender bar soap and triple-milled soap	[61]
	Liquid hand cleanser	Lavender hand-wash product	
	Shower/body cleanser	Lavender shower gel and body wash	
	Shampoo	Lavender hair shampoo	
	Conditioner	Lavender hair conditioner	
	Bath products	Bath salts; bubble bath; bath bombs	[61,62]
	Exfoliant	Lavender hand, foot and body exfoliants	[61]
	Moisturising cream	Hand, face and body creams	[160]
	Lotions and body butters	Body lotion; body butter	[61]
	Lip-care products	Lip balm; lip butter	[142]
	Skin balm/salve	Lavender skin balm/salve	
	Foot-care product	Lavender foot gel	[61]
	Deodorant	Roll-on deodorant containing lavender oil	[161]
	Perfumery	Lavender eau de toilette; cologne	[61,160]
Home fragrance and cleaning products	Scented candle	Lavender candle	[61]
	Reed diffuser	Lavender reed diffuser and refill liquid	
	Room spray	Lavender room-fragrance spray	
	Linen spray	Lavender bed-linen/linen spray	
	Scented sachet	Drawer, wardrobe and hanging sachets	[162]
	Scented drawer liner	Lavender drawer liner	[61]
	Dryer sachet	Lavender-filled dryer sachet	[162]
	Potpourri	Dried scented botanical mixtures containing lavender	[163]
	Laundry detergent	Liquid detergent containing lavender/lavandin oil	[63]
	Dishwashing cleanser	Dish soap containing lavender essential oil	[64]
	Wood-care product	Furniture polish containing beeswax and lavender oil	[65]
Decorative products, crafts and gifts	Dried bunch	Decorative lavender bouquet/bunch	[155]
	Wreath	Decorative wreath made from lavender flowers	[164]
	Lavender wand	Lavender stems woven with ribbon to make a “lavender wand”	[165]
	Natural confetti	Wedding confetti made from dried lavender flowers	[166]
	Resin jewellery	Pendant containing embedded dried lavender flowers	[167]
	Functional gift sachet	Lavender sachet in bookmark form	[62]
Aromatherapy, relaxation and filled textile products	Massage oil	Lavender massage oil	[61]
	Eye pillow	Eye pillow filled with lavender and flaxseed	[162]
	Relaxation pillow	Lavender-containing therapy pillows and other filled pillows	
	Neck/shoulder product	Lavender neck pillow or neck wrap	
	Heat pack	Heat pads/wraps filled with lavender and wheat	[61]
	Pre-sleep spray	“Slumber spray” marketed for use before sleep	
	Topical gel and rub	“Slumber gel”, “soothing gel”, and “muscle rub”	
Medical preparations and biomedical products	Oral oil capsule	Silexan capsules containing standardised lavender oil	[109]
	Oral herbal product/supplement formulation	Solid oral products containing Lavandula species; regulatory status varies by country	[168]
	Nanofibrous wound dressing	Electrospun dressing containing alginate and lavender oil	[169]
	Composite wound dressing	Polyurethane nanofibre dressing containing lavender oil and silver nanoparticles	[170]
	Nanoemulsion burn dressing	Dressing containing lavender oil in nanoemulsion form	[170]
Pet care products	Dog shampoo	Dog-care shampoo containing lavender oil	[62]
	Pet skin and coat cleanser	Shampoo listing lavender flower oil among its ingredients	[171]
Insect repellents and agricultural pest-control formulations	Moth-repellent sachet	“Moth Away Sachet”	[162]
	Commercial repellent blend containing lavender	MyggA natural formulation examined in the study	[172]
	Gel against mosquito larvae	Formulations comparing lavender extract, essential oil and gel forms	[173]
	Agricultural biopesticide prototype	Lavender-oil nanoemulsion and chitosan nanocomposite	[174]
Packaging, technical textiles, biomaterials and products derived from lavender waste	Functional textile	Fabric treated with lavender-oil microcapsules and investigated for sportswear and everyday clothing	[175]
	Active packaging paper	Packaging paper treated with lavender essential oil	[176]
	Bioplastic packaging film	Film containing PVA, tapioca starch, lavender oil and β-cyclodextrin	[177]
	Nanofibrous technical surface	Polylactic-acid nanofibrous mats loaded with lavender oil	[178]
	Particleboard	Board produced from lavender stems and Turkish red-pine chips	[179]
	Pulp and paper	Paper produced from lavender stems/secondary biomass	[180]
	Fuel pellet	Fuel pellets made from a mixture of lavender waste and pine wood	[181]
	Biochar	Material produced by carbonising lavender agricultural waste	[182]
	Modified carbon material	Zinc-modified lavender-derived biochar	
	Biotechnological intermediate products	Fungal enzymes and valuable compounds produced from distilled lavender/lavandin straws	[183]
	Compost input	Post-distillation stems used for composting	

## Data Availability

No new data were created in this study. Data sharing is not applicable to this article.
